# Predictors of emotional well-being in university professors using Machine Learning

**DOI:** 10.12688/f1000research.177439.1

**Published:** 2026-04-07

**Authors:** Rufina Narcisa Bravo-Alvarado, Juan Francisco Peraza-Garzón, Narcisa Isabel Cordero-Alvarado, Isabel Dafne Dalila Márquez-Galarza, Raúl Alberto Rengifo-Lozano, Ángel Ramón Sabando-García, Cisaddy Samantha Lazo-Bravo, Jimmy Manuel Zambrano-Acosta, Jenniffer Sobeida Moreira-Choez

**Affiliations:** 1 Facultad de Posgrado, Universidad Estatal de Milagro, Milagro, Guayas, 091050, Ecuador; 2 Facultad de Informática Mazatlán, Universidad Autonoma de Sinaloa, Culiacán, Sinaloa, 80000, Mexico; 3 Universidad Estatal de Milagro, Milagro, Guayas, 091050, Ecuador; 4 Ministerio de Educación (MINEDU) del Perú, Lima, Perú, 15021, Peru; 5 Universidad Nacional Mayor de San Marcos, Lima District, Lima Region, 15081, Peru; 6 Departamento de Matemáticas y Estadística, Pontificia Universidad Católica del Ecuador- Sede Santo Domingo, Santo Domingo, Santo Domingo, 230207, Ecuador; 7 Facultad de Posgrado, Universidad Técnica de Manabí, Portoviejo, Manabí, 130105, Ecuador

**Keywords:** university faculty, emotional well-being, prediction, artificial intelligence, machine learning, emotional regulation, socio-emotional competencies, predictive models.

## Abstract

**Background:**

In the current context of increasing psycho-emotional strain within higher education, the emotional well-being of university faculty has become a strategic variable for institutional management. In response to this challenge, the present study aimed to predict emotional well-being among faculty members from the State University of Milagro and the Technical University of Manabí through the application of machine learning algorithms.

**Methodology:**

A quantitative, explanatory, and correlational-predictive design was adopted, using a stratified probabilistic sample of 1,470 university professors. Data were collected through a psychometrically validated questionnaire and analyzed using supervised learning models implemented in Orange Data Mining.

**Results:**

The findings revealed that Gradient Boosting, Random Forest, and Neural Network algorithms achieved the highest predictive performance, reaching optimal levels of accuracy, sensitivity, and calibration. The most significant predictors identified were emotional regulation, social competencies, emotional autonomy, and emotional awareness, whereas life and well-being competencies did not show a positive relationship. Additionally, age and level of academic training were associated with higher levels of emotional well-being.

**Conclusion:**

The results highlight the capacity of machine learning algorithms to predict faculty emotional well-being with high accuracy and underscore their usefulness as decision-support tools for institutional management in occupational mental health.

## Introduction

I In recent years, interest in modeling and predicting emotional well-being across different populations has gained prominence in multidisciplinary research, particularly within the university context, where faculty members face increasing job demands, institutional instability, and diverse psychosocial stressors that may negatively affect their emotional well-being ( Hammoudi et al., 2023; Stan, 2022). In this regard, prior empirical evidence has highlighted the relevance of emotional education and its relationship with psychological well-being in higher education settings. Specifically, a comparative study conducted with graduate students demonstrated that dimensions such as emotional attention, clarity, and repair are significantly influenced by demographic and academic variables, underscoring the complexity of emotional processes within academic environments ( Moreira-Choez et al., 2024).

This phenomenon has encouraged the use of advanced analytical tools, such as machine learning (ML) algorithms, which enable the identification of complex patterns and the prediction of latent variables from large and multidimensional datasets ( Ge, 2018; Mirza et al., 2019; Rahman et al., 2022). The incorporation of ML techniques therefore represents a robust methodological approach for addressing the multifactorial nature of emotional well-being in higher education contexts.

Several studies have shown that ML models can outperform traditional statistical approaches by providing more accurate predictions of subjective well-being based on sociodemographic, psychological, and contextual data ( Ku & Min, 2024; Narayanan et al., 2025). Research conducted by Oparina et al. (2025) demonstrated that models such as k-nearest neighbors (kNN), Random Forest (RF), and neural networks achieve superior accuracy, sensitivity, and specificity metrics compared with logistic regression, thereby establishing an upper bound for the predictability of emotional well-being scores. Similarly, Kordbagheri et al. (2025) identified that traits such as agreeableness, conscientiousness, and emotional stability significantly influence academic and psychosocial outcomes and are captured with high precision by algorithms such as kNN and RF.

Recent advances in machine learning (ML) have demonstrated its potential for improving model selection and predictive accuracy in educational and psychological research. For instance, King et al. (2024) employed the scikit-learn package to implement tree-based algorithms such as Gradient Boosting Decision Trees (GBDT), AdaBoost, ExtraTrees, and LightGBM, showing that the combined use of evaluation metrics including Mean Squared Error (MSE), Mean Absolute Error (MAE), and the coefficient of determination (R ^2^) allows for the identification of the most robust predictive models based on the behavior of explanatory variables.

Despite these methodological advances, important gaps remain in the literature. First, many studies have focused predominantly on students or clinical populations, largely overlooking university faculty, a group particularly exposed to emotional exhaustion and occupational stress. Second, a substantial body of research has concentrated on predicting stress- or depression-related outcomes, without adopting a comprehensive, positive, and multidimensional perspective of emotional well-being ( Ansari et al., 2025; Thomas et al., 2007). Finally, empirical evidence on the application of ML models in Latin American educational contexts remains limited, constraining the generalizability of findings and the development of context-sensitive interventions ( Drira et al., 2024).

Within this framework, it is pertinent to conduct a study aimed at identifying the main predictors of emotional well-being among university faculty through the implementation of machine learning algorithms. This methodological strategy introduces an innovative perspective into psychoeducational research by integrating artificial intelligence techniques for the analysis of complex and multivariate data, enabling the development of robust predictive models that support evidence-based decision-making. Moreover, this approach contributes to strengthening institutional work environments and promoting faculty well-being from a preventive and systemic perspective ( Moreira-Choez et al., 2025).

Based on the problem outlined, the following research question is proposed: How do machine learning algorithms predict emotional well-being among faculty members at the State University of Milagro and the Technical University of Manabí? In response to this question, the following research hypotheses are formulated:

H1. Machine learning algorithms predict university faculty emotional well-being with high accuracy.

H2. Emotional regulation constitutes the main predictor of faculty emotional well-being.

H3. Social competencies are significantly associated with emotional well-being.

H4. Emotional autonomy and emotional awareness exert a significant influence on emotional well-being.

H5. Life and well-being competencies do not maintain a positive association with emotional well-being.

H6. Age and level of academic training significantly predict perceived emotional well-being.

H7. Gradient Boosting, Random Forest, and Neural Network models exhibit the best predictive performance.

To address the research question, the objective of the study was to predict emotional well-being among faculty members at the State University of Milagro and the Technical University of Manabí through the application of machine learning algorithms, with the aim of identifying the most influential sociodemographic and emotional factors, optimizing institutional decision-making, and guiding intervention strategies that promote faculty well-being in the university context. This methodological approach enabled the development of robust, evidence-based predictive models capable of adding value to the planning of educational policies focused on faculty mental health.

## Methods

The present study was conducted under a quantitative approach, which enabled an objective and systematic examination of the relationship between sociodemographic variables and the emotional well-being of university faculty through advanced statistical techniques. This approach was appropriate for identifying empirical regularities, estimating the magnitude of associations between variables, and developing predictive models based on structured data. Furthermore, the adoption of machine learning tools made it possible to analyze complex and non-linear patterns, overcoming the limitations of traditional statistical methods and strengthening both the explanatory and predictive capacity of the study.

Regarding the type of research, a non-experimental design was adopted, as the independent variables were not deliberately manipulated but observed in their natural context. From a methodological perspective, the study was positioned at an explanatory level, as it aimed to identify the main predictors of faculty emotional well-being and to explore relationships of influence among the analyzed variables using predictive models. The design was cross-sectional correlational, since data collection was conducted at a single point in time and the analysis focused on establishing statistical associations between sociodemographic variables and the dimensions of emotional well-being, without attempting to infer temporal changes.

The study population consisted of university faculty members from two higher education institutions in Ecuador: the State University of Milagro and the Technical University of Manabí. A stratified probabilistic sampling strategy was employed, resulting in a total sample of 1,470 participants, proportionally selected according to the population distribution of each university. To ensure sample representativeness, sex, age, and level of academic training were used as stratification criteria, allowing the heterogeneity of the faculty population to be captured and reducing potential selection bias. The detailed distribution of the sample is presented in Table 1, which shows an adequate balance across the defined strata.

**Table 1.** Sociodemographic variables of university faculty.

Variable	Category	Frequency	Percentage (%)
**Sex**	Male	594	40.4
	Female	876	59.6
	Total	1,470	100.0
**Age**	20–30 years	78	5.3
	31–40 years	492	33.5
	41–50 years	456	31.0
	51 years or older	444	30.2
	Total	1,470	100.0
**University**	Technical University of Manabí	570	38.8
	State University of Milagro	900	61.2
	Total	1,470	100.0
**Level of education**	Master’s degree	1,068	72.7
	PhD (Doctorate)	312	21.2
	Bachelor’s degree	48	3.3
	Postdoctoral training	42	2.9
**Total**		**1,470**	**100.0**

Table 1 reveals a sociodemographic composition of university faculty characterized by a higher female representation, accounting for 59.6% of the sample, reflecting a trend consistent with the progressive feminization of university teaching in Latin American contexts. In terms of age, the dominant group comprises faculty aged 31–40 years (33.5%), followed by those aged 41–50 years (31.0%) and those aged 51 years or older (30.2%), suggesting a predominantly adult academic workforce with consolidated professional trajectories. This aspect is particularly relevant for the analysis of emotional well-being, as it is commonly associated with increased workload demands and academic responsibilities. From an institutional perspective, a greater proportion of participants belonged to the State University of Milagro (61.2%) compared with the Technical University of Manabí (38.8%), allowing for organizational diversity within the Ecuadorian higher education system. Regarding educational attainment, a substantial predominance of faculty holding master’s degrees (72.7%) was observed, followed by doctoral degrees (21.2%), while bachelor’s and postdoctoral levels accounted for smaller proportions, indicating a high level of academic capital within the sample.

### Procedure for instrument administration

Prior to the administration of the data collection instrument, an informed consent process was implemented in order to ensure compliance with ethical principles of autonomy, voluntariness, and confidentiality in research involving human participants. The informed consent was administered digitally through the Google Forms platform, where participants were provided with clear and detailed information regarding the objectives of the study, its academic nature, the exclusive scientific use of the data, and the guarantees of anonymity and confidentiality of the information collected.

Acceptance of the informed consent constituted a mandatory requirement for participation in the study. Only participants who explicitly indicated their agreement were allowed to access and complete the questionnaire. In cases where informed consent was not granted, the system automatically restricted access to the form, thereby preventing participation and the recording of responses. This procedure ensured that participant inclusion was conducted in a strictly voluntary and ethically sound manner.

Once informed consent was accepted, participants proceeded to complete the emotional well-being questionnaire, which was administered using a self-report format. The instrument assessed emotional well-being from a multidimensional perspective and was structured into the following dimensions: emotional awareness, emotional regulation, emotional autonomy, social competencies, and life and well-being competencies, in addition to an overall emotional well-being dimension.

Each dimension consisted of a set of items formulated using a Likert-type scale, with graduated response options reflecting the level of agreement or perceived frequency reported by participants. The rating scale allowed for the quantification of the degree of development of each dimension, where higher scores indicated greater levels of emotional competence and perceived well-being. The instrument demonstrated adequate psychometric properties as reported in previous studies, which supported its validity and reliability for assessing emotional well-being in university populations.

**Figure 1.**
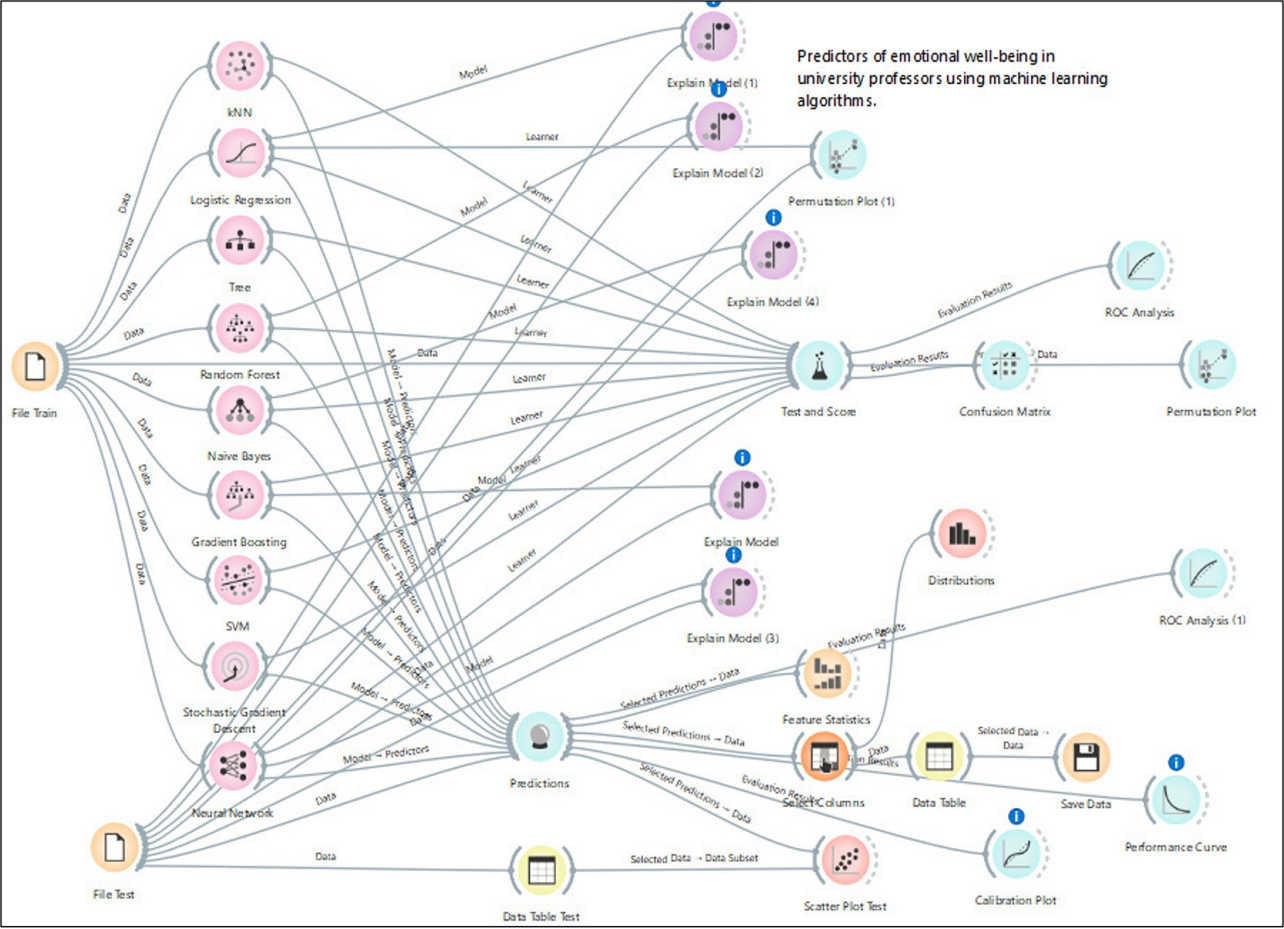
Workflow for predicting faculty emotional well-being using machine learning.

Figure 1 presents the workflow designed within the visual environment of Orange Data Mining, used for predicting emotional well-being among university faculty through the application of machine learning algorithms. This diagram graphically represents the stages of data processing, predictive model configuration, validation procedures, and result interpretation through a set of interconnected analytical modules.

### Ethical considerations

This study was conducted in accordance with established ethical principles governing research involving human participants. Prior to participation, all individuals received clear and comprehensive information about the aims, procedures, and scope of the study, and provided informed consent. Participation was entirely voluntary, and participants were informed of their right to withdraw from the study at any stage without any negative consequences.

To protect participants’ privacy, strict confidentiality measures were applied throughout the research process. All personal and identifying information was removed during data processing, and the dataset used for analysis was fully anonymized, ensuring that individual participants could not be identified. The data were accessed exclusively by the research team and were used solely for academic and scientific purposes.

The study protocol was reviewed and approved by the Institutional Review Board (IRB) of Milagro State University under approval Oficio Nro. UNEMI-VICEINVYPOSG-DP-576-2025-OF, issued on June 26, 2025, confirming compliance with ethical standards for research involving human subjects.

## Results and discussion

The results obtained in the present study make it possible to examine the relationship between university faculty emotional well-being and various sociodemographic variables. This section presents the findings derived from inferential statistical analysis aimed at identifying significant differences between levels of emotional well-being (high and low) according to sex, age, institutional affiliation, and level of academic training. For this purpose, the chi-square test was used as the criterion for statistical significance.

Table 2 is presented below, summarizing the frequencies and percentages of faculty members with high and low emotional well-being, disaggregated by the sociodemographic variables considered, together with the corresponding chi-square (χ ^2^) statistics and significance (p) values for each comparison.

**Table 2.** Relationship between emotional well-being and sociodemographic variables in university faculty.

Variable	Emotional well-being	Total	χ ^2^	p
Sex	Low	High		
Male	228	366	594	1.696
	15.5%	24.9%	40.4%	
Female	366	510	876	
	24.9%	34.7%	59.6%	
**Age**				47.253
20–30 years	18	60	78	
	1.2%	4.1%	5.3%	
31–40 years	168	324	492	
	11.4%	22.0%	33.5%	
41–50 years	240	216	456	
	16.3%	14.7%	31.0%	
51–60 years or older	168	276	444	
	11.4%	18.8%	30.2%	
**University**				0.161
Technical University of Manabí	234	336	570	
	15.9%	22.9%	38.8%	
State University of Milagro	360	540	900	
	24.5%	36.7%	61.2%	
**Level of academic training**				42.190
Master’s degree	408	660	1,068	
	27.8%	44.9%	72.7%	
PhD (Doctorate)	144	168	312	
	9.8%	11.4%	21.2%	
Bachelor’s degree	36	12	48	
	2.4%	0.8%	3.3%	
Postdoctoral training	6	36	42	
	0.4%	2.4%	2.9%	

Note. Frequencies, percentages, and chi-square test results describing the association between emotional well-being and sociodemographic variables in university faculty are reported.

Table 2 shows the relationship between emotional well-being and sociodemographic variables among university faculty. When these findings are contrasted with the available scientific literature, several convergences and divergences can be identified. First, the absence of statistically significant differences in emotional well-being by sex (p = 0.193) is consistent with studies that have not found a clear association between gender and well-being levels. However, other reports, such as Hidalgo-Andrade et al. (2021), indicate that female faculty members may exhibit greater emotional vulnerability due to differences in coping strategies and family-related demands. This highlights that the impact of gender may vary according to cultural and institutional context ( Mendes Rodrigues et al., 2019).

Regarding age, a significant association was identified (p < 0.001). Higher levels of well-being in the 31–40 and 51–60 age groups may be related to greater consolidation of academic careers and the development of socio-emotional competencies. Research such as that by Pace et al. (2021) suggests that university workload and bureaucratic demands tend to be better managed by faculty with accumulated experience, thereby reducing perceptions of discomfort and stress. Nevertheless, in the 41–50 age group, the predominance of low well-being (16.3%) may be associated with the overload of administrative, family, and research responsibilities, a phenomenon also documented in studies on burnout in higher education ( Barreto et al., 2023).

With respect to the institutional environment, the analysis did not reveal differences between universities (p = 0.689). Although this result suggests that emotional well-being does not depend on institutional affiliation, studies such as Gebisa et al. (2020) emphasize that organizational policies, working conditions, and institutional support significantly influence occupational stress and faculty emotional health. This indicates that, despite the absence of differences in this study, it is necessary to further explore specific institutional variables such as psychosocial support programs and employment conditions.

Finally, the level of academic training showed statistically significant differences (p < 0.001). Faculty members holding master’s and postdoctoral degrees exhibited higher levels of emotional well-being, suggesting that advanced training not only provides professional competencies but also access to academic support networks that facilitate stress coping. Studies such as Barreto et al. (2022) indicate that academic overexertion and workaholism are associated with burnout; however, strong professional preparation can mitigate these effects when accompanied by institutional support strategies. Likewise, Xu and Wang (2023) demonstrate that stress related to research, teaching, and administrative management is linked to lower life satisfaction, mediated by burnout, underscoring the importance of balancing work demands with personal and academic development.

Table 3 presents the Pearson correlation coefficients between emotional well-being and its associated dimensions, with the aim of examining the strength and direction of the relationships among the emotional variables assessed in the faculty population. This analysis makes it possible to identify the emotional factors that are significantly associated with perceived well-being, providing relevant empirical evidence for understanding the mechanisms that underpin it and enabling the design of interventions aimed at strengthening these dimensions in educational contexts.

**Table 3.** Correlations among dimensions of emotional well-being in faculty.

Variables	Emotional well-being	Emotional awareness	Emotional regulation	Emotional autonomy	Social competencies	Life and well-being competencies
Emotional Well-being	1	.592**	.832**	.623**	.749**	−0.005**
Emotional awareness	.592**	1	.448**	.279**	.301**	−.082**
Emotional regulation	.832**	.448**	1	.198**	.652**	−.401**
Emotional autonomy	.623**	.279**	.198**	1	.296**	.332**
Social competencies	.749**	.301**	.652**	.296**	1	−.286**
Life and well-being competencies	−0.005	−.082**	−.401**	.332**	−.286**	1

Note. Correlations are significant at the 0.01 level (two-tailed).

Table 3 identifies meaningful correlations between emotional well-being and its associated dimensions among university faculty, allowing for a comprehensive view of how emotional and social competencies relate to psychological well-being. The results show that emotional regulation exhibits the strongest association with emotional well-being (r = .832), which is consistent with international evidence highlighting the ability to manage and modulate emotions as a key predictor of faculty well-being. Ismail et al. (2023) report that emotional regulation, together with self-efficacy and professional identity, directly influences the psychological well-being of language teachers, reinforcing the notion that this competence is essential in demanding educational contexts.

Similarly, social competence shows a high correlation (r = .749), supporting the literature that positions positive interpersonal interaction as a protective factor against occupational stress. Carstensen and Klusmann (2021) demonstrated that social competence negatively predicts emotional exhaustion among pre-service teachers, functioning as an adaptive resource during professional transition. Complementarily, Jennings (2016) explains in the prosocial classroom model that teachers’ socioemotional competence contributes not only to their own well-being but also to a positive classroom climate.

Emotional autonomy, with a moderate correlation (r = .623), underscores the importance of affective independence and self-efficacy. Collie et al. (2024) found that perceived autonomy in learning enhances overall socioemotional competence and, consequently, increases well-being levels, confirming the value of this dimension as a foundation of emotional balance. With regard to emotional awareness, although its correlation was lower (r = .592), it remains significant, confirming its role as an initial stage in the development of socioemotional competencies. Recent studies among postgraduate students indicate that emotional clarity and attention to one’s own emotions vary with age, influencing emotional repair capacity and affective stability ( Aragundi-Moncada et al., 2025).

Finally, the negative correlation between life and well-being competencies and most dimensions, particularly emotional regulation (r = −.401), is noteworthy. This apparent contradiction may be related to differences in construct operationalization, as research such as Etchebehere et al. (2024) shows that when teachers promote autonomy and emotional regulation in their students, they tend to experience higher levels of occupational well-being. This suggests that the observed dissociation may be attributable to limitations of the instrument used rather than to the absence of a substantive relationship.

Table 4 presents the performance of different machine learning algorithms in predicting the level of emotional development (high and low) among university faculty. For each model, key evaluation metrics are reported, including the Area Under the Curve (AUC), Overall Accuracy (CA), F1-score, Precision, Recall (Sensitivity), and the Matthews Correlation Coefficient (MCC). This comparative analysis makes it possible to identify the models with the greatest discriminative capacity and predictive robustness, thereby facilitating the selection of the most efficient algorithms for the automated assessment of faculty emotional development.

**Table 4.** Performance metrics of predictive models.

Emotional development – low						
Model	AUC	CA	F1	Prec	Recall	MCC
Logistic regression	1.000	1.000	1.000	1.000	1.000	1.000
Tree	0.984	0.975	0.969	0.962	0.976	0.948
Gradient boosting	1.000	1.000	1.000	1.000	1.000	1.000
Random forest	1.000	1.000	1.000	1.000	1.000	1.000
kNN	1.000	1.000	1.000	1.000	1.000	1.000
SVM	1.000	0.995	0.993	0.988	0.998	0.989
SGD	1.000	1.000	1.000	1.000	1.000	1.000
Neural network	1.000	1.000	1.000	1.000	1.000	1.000
Naive bayes	0.961	0.893	0.870	0.854	0.887	0.780
Emotional development – high						
Model	AUC	CA	F1	Prec	Recall	MCC
Logistic regression	1.000	1.000	1.000	1.000	1.000	1.000
Tree	0.984	0.975	0.979	0.984	0.974	0.948
Gradient boosting	1.000	1.000	1.000	1.000	1.000	1.000
Random forest	1.000	1.000	1.000	1.000	1.000	1.000
kNN	1.000	1.000	1.000	1.000	1.000	1.000
SVM	1.000	0.995	0.995	0.999	0.992	0.989
SGD	1.000	1.000	1.000	1.000	1.000	1.000
Neural network	1.000	1.000	1.000	1.000	1.000	1.000
Naive bayes	0.961	0.893	0.909	0.921	0.897	0.780

Note. Performance metrics of predictive models for low and high emotional development levels are presented. Reported measures include AUC, CA, F1, precision, recall, and MCC.

The results presented in Table 4 indicate outstanding performance for most predictive models applied to faculty emotional development, with perfect metrics for accuracy, recall, and F1-score observed in algorithms such as Logistic Regression, Random Forest, Gradient Boosting, kNN, SGD, and Neural Networks. While these results reflect an optimal fit to the training data, they also suggest a potential risk of overfitting, as noted by Pal and Patel (2020), who caution that models may learn sample-specific patterns and consequently lose generalization capacity when applied to unseen data.

The comparatively lower performance of the Naive Bayes model at both levels of emotional development (AUC = 0.961; F1 = 0.870 for the low level) confirms the limitations of this algorithm in contexts where predictor variables exhibit high collinearity, a common characteristic in psychoeducational research. This finding is consistent with Saif et al. (2024), who reported that Naive Bayes is frequently outperformed by more robust classifiers, such as SVM and Random Forest, particularly when complex dependencies among attributes are present.

In contrast, the performance of the SVM model, although slightly lower than that of ensemble methods and neural networks (Accuracy = 0.995), remained at highly satisfactory levels. Nandi et al. (2021) demonstrated that combining SVM with stream learning techniques enhances emotional classification in e-learning contexts, highlighting its applicability in dynamic environments. Regarding ensemble algorithms, particularly Random Forest and Gradient Boosting, the results confirm their potential as robust and stable methods for psychological prediction tasks. Chutia and Baruah (2024) further emphasize that ensemble learning and deep learning approaches represent some of the most promising trends in emotion analysis, especially when integrated with architectures such as BERT or Bi-LSTM to improve the interpretation of latent variables in well-being research.

**Figure 2.**
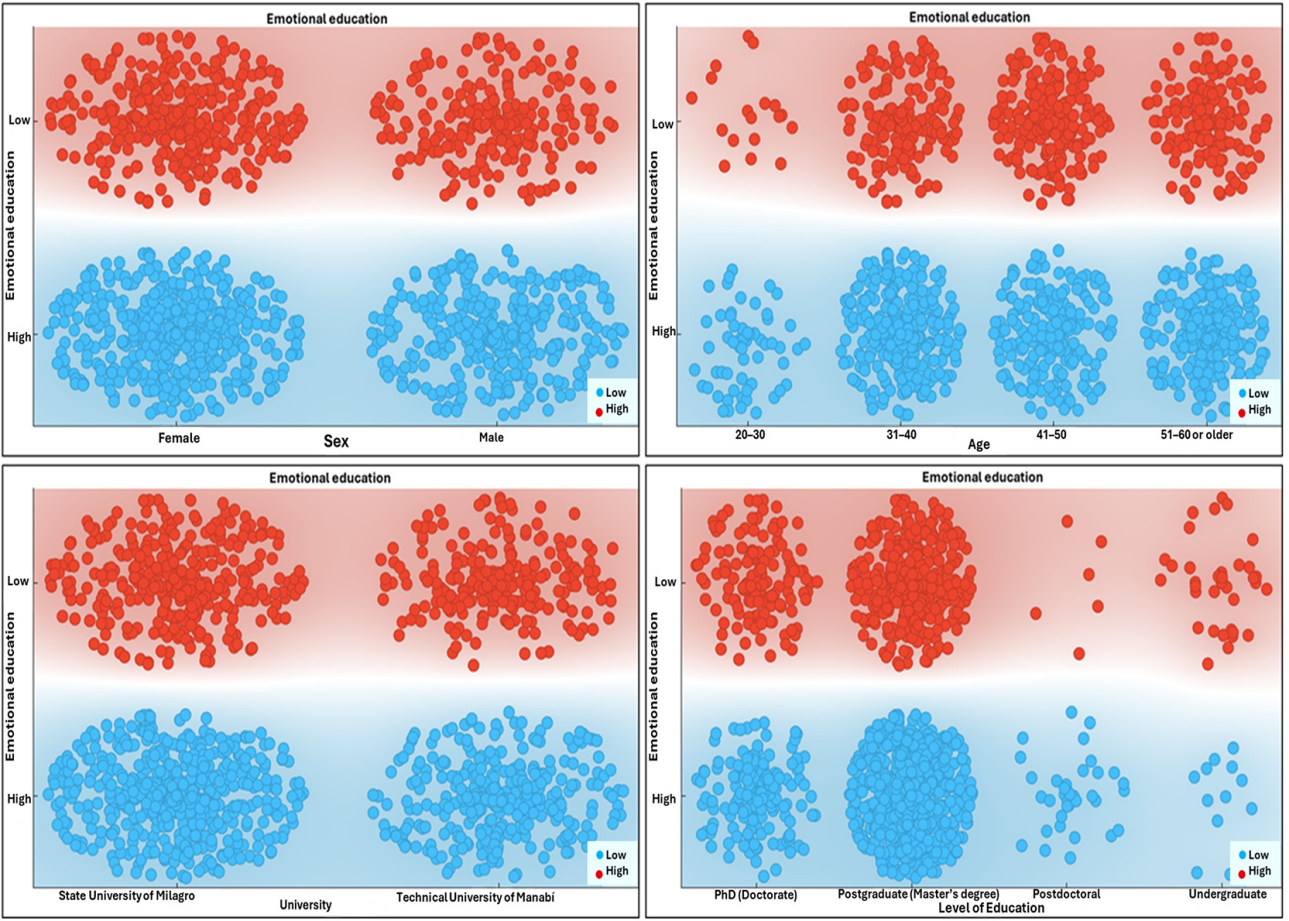
Emotional education behavior in relation to sociodemographic conditions. Note. Scatter plots depict the distribution of observations across sociodemographic conditions in relation to emotional education behavior. Background shading indicates the classification regions learned by the model.

Figure 2 shows a visual representation of the behavior of emotional education in relation to specific sociodemographic variables within the faculty sample. Using scatter plots generated through machine learning algorithms, levels of emotional development are contrasted with sex, age, institutional affiliation, and level of academic training. This visualization makes it possible to identify distribution patterns that reveal potential associations between personal and educational factors and the emotional status of university faculty.

In Figure 2, which illustrates the behavior of emotional development in contrast with sociodemographic conditions, relevant differences are observed that confirm the trends identified in the previous tables. A higher concentration of faculty members with high emotional development is found among women, within the 31–40 age group, and among those holding postgraduate qualifications. These patterns are consistent with research highlighting the influence of sociodemographic and professional characteristics on emotional intelligence and faculty well-being. In this regard, the study by Arteaga-Cedeño et al. (2022) demonstrated that gender, age, and educational level exert a significant effect on teachers’ socioemotional competencies, with women and individuals with higher academic training reporting greater levels of emotional perception and regulation. Similar findings were reported by Pérez-Díaz et al. (2022), who, in examining the invariance of the emotional intelligence construct in clinical and educational populations, identified educational attainment as a key differentiating factor in explaining emotional competence levels and their impact on well-being.

Conversely, the relative homogeneity observed in the figure with respect to institutional affiliation suggests that the institutional context alone does not guarantee the strengthening of emotional development unless it is accompanied by specific training policies. Studies such as that of Jelińska and Paradowski (2021) indicate that, in high-pressure contexts such as remote teaching during the pandemic, institutional conditions may moderate faculty well-being; however, it is the interaction between job demands and personal resources that more strongly determines emotional adaptation. Complementarily, Casacchia et al. (2021) found that distance education policies implemented during the pandemic in Italy generated high levels of emotional distress among faculty, with those lacking sustained institutional support being particularly vulnerable.

The greater presence of faculty with postgraduate and doctoral degrees within the high emotional development group may also be interpreted as an advantage derived from exposure to formative processes that foster self-awareness, self-regulation, and critical reflection. This interpretation aligns with the proposal of Abdellatif et al. (2017), who argue that emotional intelligence can be intentionally cultivated through training and development programs, with more pronounced effects among individuals with advanced academic trajectories. Likewise, recent research has emphasized that faculty with higher educational levels possess more coping resources, which facilitates a better balance between professional demands and personal life ( Coman et al., 2024).

Finally, the relationship between gender and emotional development observed in this figure is supported by longitudinal studies on mental health and emotional education. Van Vuuren et al. (2018) showed that women tend to experience higher levels of emotional problems during adolescence; however, in adulthood they develop more effective coping strategies, which translates into improved emotional management in professional contexts. This finding converges with the work of Lizana and Lera (2022), who reported that despite the substantial impact of the pandemic on faculty, women developed stronger adaptive capacities in response to stress, which may explain their higher concentration within the high emotional development group.

**Figure 3.**
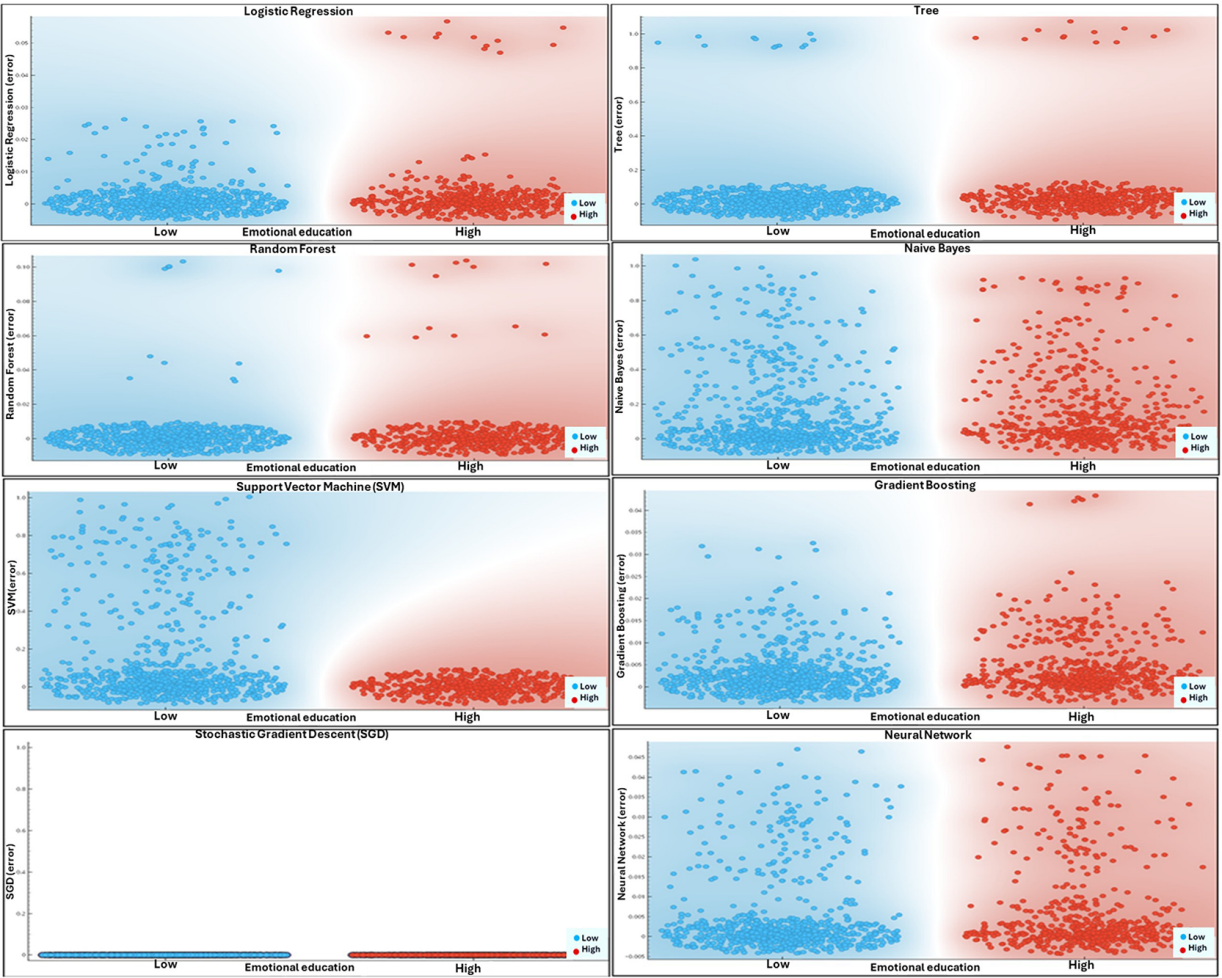
Dynamics of predictive models according to the level of emotional development. Note. Scatter plots illustrate the dynamics of the predictive models across different levels of emotional development. Each panel corresponds to a specific level, showing the distribution of instances by class, while the background color gradient represents the model’s decision boundary.

Figure 3 represents the classification dynamics generated by various machine learning algorithms according to the level of emotional development among university faculty. Each subplot illustrates how the models separate classes corresponding to high and low levels of emotional development through decision boundaries projected onto a two-dimensional plane resulting from dimensionality reduction. This visualization allows for the assessment of each model’s discriminative capacity and the observation of class overlap, a key aspect in evaluating predictive quality.

Based on Figure 3, the dynamics of the predictive models indicate that the level of emotional development introduces a differentiating structure in the classification capacity of the algorithms employed. The progressive separation between classes and the reduction of overlapping regions suggest that the models capture consistent and non-trivial emotional patterns, indicating effective learning of latent regularities. This behavior is consistent with studies showing that negative academic emotions can be predicted with greater accuracy when individual and contextual variables are integrated through machine learning techniques, particularly in ensemble models such as Random Forest, which tend to outperform traditional linear approaches ( Ma et al., 2025). In this context, the visualization provides empirical support for the usefulness of predictive modeling in representing the complexity of emotional development from a non-reductionist perspective.

Complementarily, the heterogeneous dispersion of the data suggests that emotional development behaves as a dynamic process characterized by variability and instability rather than by stable states. This interpretation aligns with longitudinal research demonstrating that emotional variability and instability, in both positive and negative affect, are relevant predictors of psychological distress and depressive symptoms in educational populations ( Yang et al., 2025). The ability of the models to adapt to non-linear relationships allows these temporal fluctuations to be captured, which often remain undetected in descriptive or cross-sectional analyses. Consequently, the observed dynamics reinforce the need to employ predictive approaches that take into account the temporal and contextual nature of emotions in educational settings.

Finally, the interpretation of these findings is strengthened when contrasted with studies that integrate socioemotional development and data science across different educational levels. Predictive models applied to cognitive and socioemotional development have been shown to achieve high levels of accuracy when simultaneously incorporating personal and environmental factors, enabling the anticipation of trajectories and the orientation of early interventions ( Brezov et al., 2025). Convergently, the literature in educational and organizational psychology emphasizes that emotions directly influence well-being and adaptation among both students and faculty, particularly in contexts of high demand or institutional change ( Čábelková et al., 2022).

**Figure 4.**
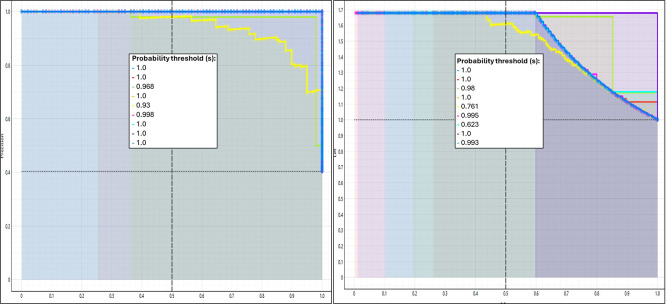
Performance curves of learning algorithms according to emotional development level. Note. This figure shows the performance curves of the learning algorithms across different probability thresholds, stratified by emotional development level. Colored lines represent the evaluated thresholds, and dashed lines indicate reference values used for comparison.

Figure 4 presents the comparative performance curves of the machine learning algorithms applied to the prediction of emotional development levels in university faculty. These curves make it possible to assess the generalization capacity of each model under different conditions by observing their behavior across successive iterations or variations in the proportion of data used for training and validation. The visualization synthesizes accumulated metrics such as accuracy, sensitivity, and the operational stability of the models.

Based on Figure 4, the performance curves show that the behavior of the learning algorithms varies systematically according to the level of emotional development, suggesting a direct relationship between emotional stability and the predictive capacity of the models. In the initial stages, an accelerated learning phase is observed, followed by stabilization phases in which performance metrics tend to saturate, a pattern commonly found in models trained with complex affective variables. This behavior has been documented in studies that incorporate emotions as inputs in machine learning processes, reporting that the inclusion of emotional states improves model accuracy and generalization in digital educational contexts ( Du et al., 2023). In this sense, the curves reflect not only computational efficiency but also the sensitivity of the algorithms to capture meaningful emotional regularities during training.

Complementarily, the divergence observed among algorithms in the later stages of training suggests differences in their capacity to adapt to emotional variability. Some models maintain stable performance in the face of progressive changes in the data, whereas others exhibit declines associated with overfitting or reduced generalization ability. This finding is consistent with research highlighting that neural network–based approaches and ensemble methods offer advantages when emotional variables are integrated, due to their ability to model non-linear relationships and temporal dynamics ( Xiao et al., 2022). Likewise, studies focused on emotion recognition through physiological signals have shown that performance stability depends on appropriate feature selection and the algorithm employed, reinforcing the interpretation of the differences observed in the curves ( Domínguez-Jiménez et al., 2020).

Finally, a joint reading of the curves allows the inference that higher levels of emotional development are associated with more consistent and less fluctuating learning trajectories, supporting the hypothesis that the emotional dimension acts as a modulator of algorithmic performance. Recent studies in hybrid educational environments have demonstrated that models integrating emotion detection and adaptive strategies achieve significant gains in accuracy and learning personalization, particularly when advanced techniques such as deep reinforcement learning are applied ( Govea et al., 2024).

**Figure 5.**
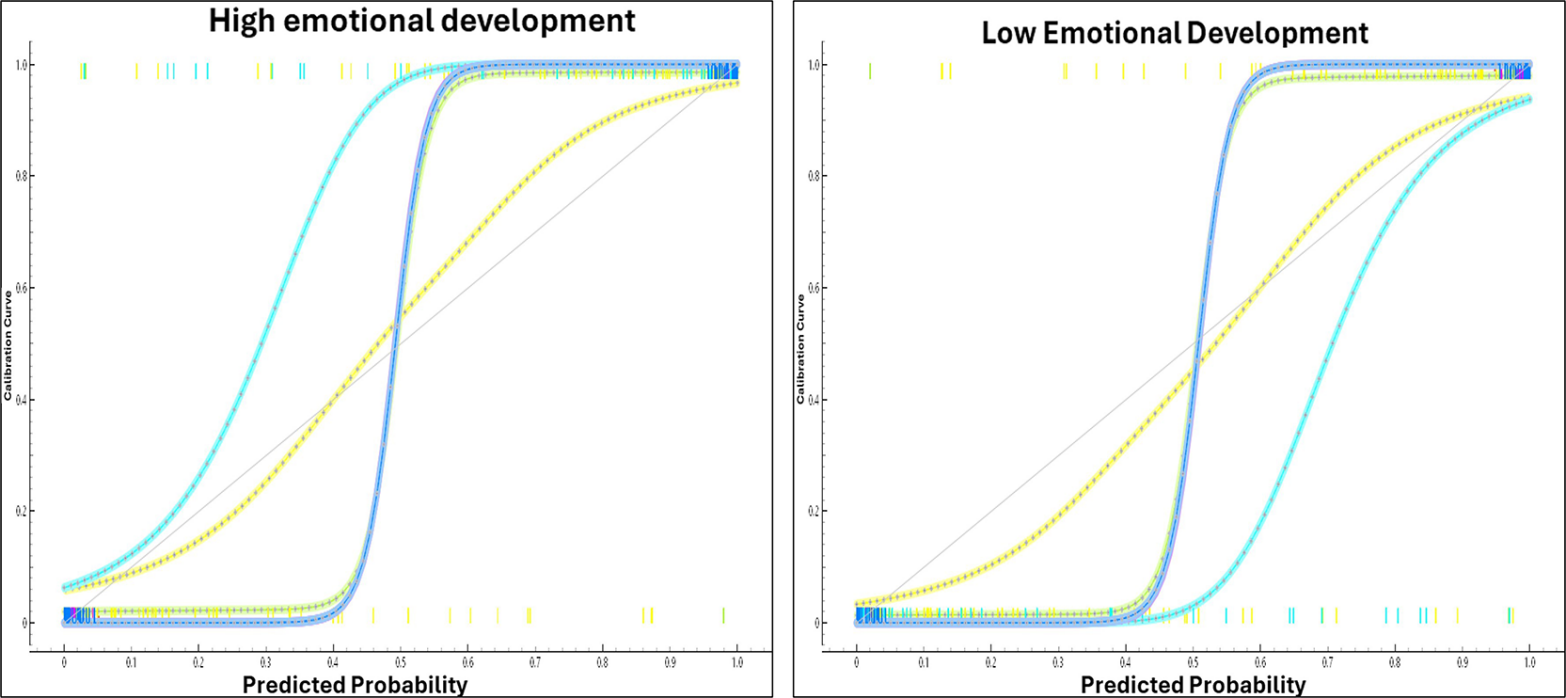
Calibration of emotional development models in university faculty. Note. The figure presents calibration curves comparing predicted probabilities and observed frequencies for the emotional development models. The reference diagonal indicates ideal calibration.

Figure 5 displays the calibration curves of the predictive models applied to the classification of emotional development in university faculty. These curves make it possible to examine the extent to which the probabilities predicted by the algorithms correspond to the frequencies observed in the real data. Adequate calibration indicates that the model not only classifies correctly but also provides reliable probabilistic estimates, which is crucial for implementation in sensitive educational contexts such as faculty emotional health.

Based on Figure 5, the calibration of emotional development models in university faculty allows the evaluation of the coherence between predicted probabilities and observed frequencies, a key aspect for ensuring valid interpretations in complex educational contexts. The curves show that some algorithms exhibit close alignment with the reference diagonal, indicating adequate calibration and, therefore, reliable estimates of emotional development levels. This behavior is consistent with the literature emphasizing the need for well-calibrated models when working with socioemotional constructs, given their multidimensional nature and sensitivity to institutional context ( Lozano-Peña et al., 2021). From this perspective, calibration reflects not only statistical fit but also the model’s ability to realistically represent faculty emotional competencies, which are closely associated with professional well-being and educational quality.

In contrast, other curves display systematic deviations, particularly in intermediate probability ranges, suggesting overestimation or underestimation of emotional development. This pattern has been described in studies warning that predictive models may be affected by biases arising from the measurement of emotional competencies and from the heterogeneity of faculty career trajectories ( Aldrup et al., 2020). Recent literature emphasizes that faculty emotional competence is not a homogeneous trait, but rather a set of skills that evolve in response to job demands, relational contexts, and emotional regulation strategies ( Savina et al., 2025). Therefore, the discrepancies observed in calibration may be interpreted as a reflection of this structural variability rather than as mere technical limitations of the model.

Finally, a critical interpretation of the calibration results gains further depth when linked to studies highlighting the role of emotional intelligence and socioemotional learning in the sustainability of faculty performance. Research has shown that faculty members with higher emotional competencies exhibit greater work engagement and lower stress impact, which indirectly influences the stability of predictive patterns ( Mérida-López et al., 2019). Likewise, recent systematic reviews in higher education indicate that integrating technological and socioemotional approaches enhances model precision and their usefulness for informing educational decision-making ( Mukhemar et al., 2025).

## Conclusions

The present study was conducted in response to growing concern regarding the emotional well-being of university faculty and the need for advanced analytical tools capable of understanding and anticipating this phenomenon from a comprehensive perspective. Using a quantitative and predictive approach, the primary objective was to predict emotional well-being in university faculty through machine learning algorithms, integrating sociodemographic variables and socioemotional dimensions. The results obtained allow this objective to be considered achieved, as the implemented models demonstrated a high capacity to identify complex and non-linear patterns associated with faculty emotional development, providing robust empirical evidence within a Latin American context that has been scarcely explored to date.

With respect to the research question, the findings confirm that machine learning algorithms can predict faculty emotional well-being with high accuracy, thereby validating the methodological approach adopted. Consistently, emotional regulation emerged as the most influential predictor, followed by social competencies, emotional autonomy, and emotional awareness, all of which showed positive and statistically significant associations with emotional well-being. In addition, age and level of academic training emerged as relevant sociodemographic variables, whereas life and well-being competencies did not show a positive relationship, suggesting potential conceptual tensions or limitations in their operationalization. At the algorithmic level, Gradient Boosting, Random Forest, and Neural Network models exhibited the strongest predictive performance; however, the presence of near-perfect metrics warrants cautious interpretation due to the potential risk of overfitting.

Nevertheless, several limitations should be acknowledged. The cross-sectional design prevents causal inference and limits the understanding of temporal changes in emotional well-being. Moreover, although the sample was large and probabilistic, it was restricted to two Ecuadorian universities, which constrains the generalizability of the findings to other institutional and cultural contexts. Additionally, the reliance on self-report instruments may introduce perceptual biases. In this regard, future research should incorporate longitudinal designs, multicenter samples, and objective or multimodal indicators, as well as external validation strategies for predictive models. Complementarily, further work is recommended on hybrid approaches that integrate machine learning techniques with socioemotional theoretical frameworks, in order to enhance model interpretability and support their application in the development of institutional policies aimed at the sustainable promotion of faculty well-being.

## Data Availability

Repository name: Figshare: Data from the article titled: Predictors of emotional well-being in university professors using Machine Learning.xlsx. DOI: https://doi.org/10.6084/m9.figshare.31130092 ( Bravo-Alvarado et al., 2026a). Repository name: figshare Questionnaire of the data from the article entitled “Predictors of emotional well-being in university professors using Machine Learning”. figshare. https://doi.org/10.6084/m9.figshare.31290157 ( Bravo-Alvarado et al., 2026b). Data are available under the terms of the Creative Commons Attribution 4.0 International license (CC-BY 4.0).
