# Supplementary material for: Predictors of emotional well-being in university professors using Machine Learning
Source: F1000Res. 2026 Apr 7;15:487. [Version 1] doi: 10.12688/f1000research.177439.1 (PMC13146459; doi:10.12688/f1000research.177439.1)
Supplement: Peer review history

# Reviewer report: Reviewer response for version 1

**DOI:** 10.5256/f1000research.195666.r474379

**Published:** 2026-05-05

**Authors:** Jose Alberto Sánchez López

**Affiliations:** 1 Instituto Tecnologico Superior de Escarcega, Escárcega, Campeche, Mexico

Overall, the research is clear and sufficient. The contribution to science is also very important. However, hypothesis H5 exhibits a significant conceptual weakness, as it is formulated negatively without explicit theoretical support to justify this directionality, which disrupts the internal coherence of the proposed model. Within the study's conceptual framework, emotional competencies are presented as factors that contribute positively to well-being, so introducing a hypothesis that denies this relationship without solid argumentation generates logical and methodological inconsistency. Furthermore, the negative formulation is closer to a null hypothesis than a testable research hypothesis, which weakens the rigor of the approach. This type of relationship, if empirically proven to be negative, should be reported as an unexpected finding in the results and discussion section, accompanied by a critical interpretation, rather than being anticipated without theoretical support in the hypothetical phase.

Is the work clearly and accurately presented and does it cite the current literature?

Yes

If applicable, is the statistical analysis and its interpretation appropriate?

Yes

Are all the source data underlying the results available to ensure full reproducibility?

Yes

Is the study design appropriate and is the work technically sound?

Yes

Are the conclusions drawn adequately supported by the results?

Yes

Are sufficient details of methods and analysis provided to allow replication by others?

Yes

Reviewer Expertise:

Administration sciences, organizational studies, and higher education research, with a focus on emotional well-being, socio-emotional competencies, and institutional performance. Expertise in quantitative methods, statistical analysis, and machine learning applications in educational and social research contexts. Additional experience in research methodology, business models, and social and solidarity economy (ESS).

I confirm that I have read this submission and believe that I have an appropriate level of expertise to confirm that it is of an acceptable scientific standard.

# Reviewer report: Reviewer response for version 1

**DOI:** 10.5256/f1000research.195666.r474373

**Published:** 2026-04-30

**Authors:** Richard Callan Bacilio

**Affiliations:** 1 University of Huanuco, Huanuco, Huanuco, Peru

It meets established scientific standards, presenting a clear methodology, consistent and reliable results, and a well-supported discussion. The writing is precise, coherent, and well-structured, facilitating comprehension of the content. For these reasons, it is suitable for indexing in a recognized academic journal and contributes valuable, relevant knowledge to its field of study.

Is the work clearly and accurately presented and does it cite the current literature?

Yes

If applicable, is the statistical analysis and its interpretation appropriate?

Yes

Are all the source data underlying the results available to ensure full reproducibility?

Yes

Is the study design appropriate and is the work technically sound?

Yes

Are the conclusions drawn adequately supported by the results?

Yes

Are sufficient details of methods and analysis provided to allow replication by others?

Yes

Reviewer Expertise:

My areas of expertise include education, management, and communication. I have experience in teaching and learning processes, organizational management, and the analysis of communication strategies. This background allows me to assess methodological rigor, clarity of presentation, and the practical relevance of research in these fields.

I confirm that I have read this submission and believe that I have an appropriate level of expertise to confirm that it is of an acceptable scientific standard.
